# Correction to “Young carers in Japan: Reliability and validity testing of the BBC/University of Nottingham young carers survey questionnaire and prevalence estimation in 5000 adolescents”

**DOI:** 10.1002/pcn5.70010

**Published:** 2024-09-08

Kanehara A, Morishima R, Takahashi Y, Koike H, Usui K, Sato SI, Uno A, Sawai Y, Kumakura Y, Yagishita S, Usami S, Morita M, Morita K, Kanata S, Okada N, Yamasaki S, Nishida A, Ando S, Koike S, Shibuya T, Joseph S, Kasai K. Young carers in Japan: reliability and validity testing of the BBC/University of Nottingham young carers survey questionnaire and prevalence estimation in 5000 adolescents. *PCN Rep*. 2022;1(3):e46. https://doi.org/10.1002/pcn5.46

In Table 2, the range indicated for the “Well‐being total score” was “0–24.” This is incorrect. It should be “0–20.”

We apologize for this error.

